# Supplementary material for: Prevalence and Genetic Diversity of Toxin Genes in Clinical Isolates of Clostridium perfringens: Coexistence of Alpha-Toxin Variant and Binary Enterotoxin Genes (bec/cpile)
Source: Toxins (Basel). 2019 Jun 6;11(6):326. doi: 10.3390/toxins11060326 (PMC6628447; doi:10.3390/toxins11060326)
Supplement: Supplementary file 1 [file toxins-11-00326-s001.zip › toxins-514923-si/R1-Figure S1-alpha-align.pdf]

Figure S1 Alignment of alpha-toxin amino acid sequences representing toxin types F, I, IVb, IVc, IVd, V, Vd, Ve, Vf, Vg, and VIII. Asterisk indicates identical amino acid. Residues that are different from those of type F are shown in red. The central loop domain which mediates interaction with ganglioside GM1a is marked in blue in the bottom line, and its key residues are indicated with vertical bars. Shadowed portion above the sequence indicates C-domain (membrane-binding region), in which critical amino acid sites for binding of toxin are marked with #.

```
                    Signal peptide
F       MKRKICKALICATLATSLWAGASTKVYAWDGKIDGTGTHAMIVTQGVSILENDLSKNEPE   60
I       MKRKICKALICAALATSLWAGASTKVYAWDGKIDGTGTHAMIVTQGVSILENDLSKNEPE   60
VIII    MKRKICKALICATLATSLWAGASTKVYAWDGKIDGTGTHAMIVTQGVSILENDLSKNEPE   60
IVd     MKRKICKALICAALATSLWAGASTKVYAWDGKIDGTGTHAMIVTQGVSILENDMSKNEPE   60
IVc     MKRKICKALICAALATSLWAGASTKVYAWDGKIDGTGTHAMIVTQGVSILENDMSKSEPE   60
IVb     MKRKICKALICAALATSLWAGASTKVYAWDGKIDGTGTHAMIVTQGVSILENDMSKNEPE   60
Vg      MKRKICKALICAALATSLWAGASTKVYAWDGKIDGTGTHAMIVTQGVSILENDLSKNEPE   60
Vd      MKRKICKALICAALATSLWAGASTKVYAWDGKIDGTGTHAMIVTQGVSILENDLSKNEPE   60
Ve      MKRKICKALICAALATSLWAGASTKVYAWDGKIDGTGTHAMIVTQGVSILENDLSKNEPE   60
V       MKRKICKALICAALATSLWAGASTKVYAWDGKIDGTGTHAMIVTQGVSILENDLSKNEPE   60
Vf      MKRKICKALICAALATSLWAGASTKVYAWDGKIDGTGTHAMIVTQGVSILENDLSKNEPE   60
        ************:****************************************:**.***

F       SVRKNLEILKENMHELQLGSTYPDYDKNAYDLYQDHFWDPDTDNNFSKDNSWYLAYSIPD   120
I       SVRKNLEILKENMHELQLGSTYPDYDKNAYDLYQDHFWDPDTDNNFSKDNSWYLAYSIPD   120
VIII    SVRKNLEILKENMHELQLGSTYPDYDKNAYDLYQDHFWDPDTDNNFSKDNSWYLAYSIPD   120
IVd     SVRKNLEILKENMHELQLGSTYPDYDKNAYDLYQDHFWDPDTDNNFSKDNSWYLAYSIPD   120
IVc     SVRKNLEILKENMHELQLGSTYPDYDKNAYDLYQDHFWDPDTDNNFSKDNSWYLAYSIPD   120
IVb     SVRKNLEILKENMHELQLGSTYPDYDKNAYDLYQDHFWDPDTDNNFSKDNSWYLAYSIPD   120
Vg      SVRKNLEILKENMHELQLGSTYPDYDKNAYDLYQDHFWDPDTDNNFSKDNSWYLAYSIPD   120
Vd      SVRKNLEILKENMHELQLGSTYPDYDKNAYDLYQDHFWDPDTDNNFSKDNSWYLAYSIPD   120
Ve      SVRKNLEILKENMHELQLGSTYPDYDKNAYDLYQDHFWDPDTDNNFSKDNSWYLAYSIPD   120
V       SVRKNLEILKENMHELQLGSTYPDYDKNAYDLYQDHFWDPDTDNNFSKDNSWYLAYSIPD   120
Vf      SVRKNLEILKENMHELQLGSTYPDYDKNAYDLYQDHFWDPDTDNNFSKDNSWYLAYSIPD   120
        ************************************************************

F       TGESQIRKFSALARYEWQRGNYKQATFYLGEAMHYFGDIDTPYHPANVTAVDSAGHVKFE   180
I       TGESQIRKFSALARYEWQRGNYKQATFYLGEAMHYFGDIDTPYHPANVTAVDSAGHVKFE   180
VIII    TGESQIRKFSALARYEWQRGNYKQATFYLGEAMHYFGDIDTPYHPANVTAVDSAGHVKFE   180
IVd     TGESQIRKFSALARYEWQRGNYKQATFYLGEAMHYFGDIDTPYHPDNVTAVDSAGHVKFE   180
IVc     TGESQIRKFSALARYEWQRGNYKQATFYLGEAMHYFGDIDTPYHPANVTAVDSAGHVKFE   180
IVb     TGESQIRKFSALARYEWQRGNYKQATFYLGEAMHYFGDIDTPYHPANVTAVDSAGHVKFE   180
Vg      TGESQIRKFSALARYEWQRGNYKQATFYLGEAMHYFGDIDTPYHPANVTAVDSAGHVKFE   180
Vd      TGESQIRKFSALARYEWQRGNYKQATFYLGEAMHYFGDIDTPYHPANVTAVDSAGHVKFE   180
Ve      TGESQIRKFSALARYEWQRGNYKQATFYLGEAMHYFGDIDTPYHPANVTAVDSAGHVKFE   180
V       TGESQIRKFSALARYEWQRGNYKQATFYLGEAMHYFGDIDTPYHPANVTAVDSAGHVKFE   180
Vf      TGESQIRKFSALARYEWQRGNYKQATFYLGEAMHYFGDIDTPYHPANVTAVDSAGHVKFE   180
        ********************************************* **************

F       TFAEERKEQYKINTAGCKTNEDFYADILKNKDFNAWSKEYARGFAKTGKSIYYSHASMSH   240
I       TFAEERKEQYKINTAGCKTNEDFYADILKNKDFNAWSKEYARGFAKTGKSIYYSHASMSH   240
VIII    TFAEERKEQYKINTAGCKTNEDFYADILKNKDFNAWSKEYARGFAKTGKSIYYSHASMSH   240
IVd     TFAEERKEQYKINTAGCKTNEDFYADILKNKDFNAWSKEYARGFAKTGKSIYYSHASMSH   240
IVc     TFAEERKEQYKINTAGCKTNEDFYADILKNKDFNAWSKEYARGFAKTGKSIYYSHASMSH   240
IVb     TFAEERKEQYKINTAGCKTNEDFYADILKNKDFNAWSKEYARGFAKTGKSIYYSHASMSH   240
Vg      TFAEERKEQYKINTAGCKTNEAFYTDILKNKDFNAWSKEYARGFAKTGKSIYYSHASMSH   240
Vd      TFAEERKEQYKINTAGCKTNEAFYTDILKNKDFNAWSKEYARGFAKTGKSIYYSHASMSH   240
Ve      TFAEERKEQYKINTAGCKTNEAFYTDILKNKDFNAWSKEYARGFAKTGKSIYYSHASMSH   240
V       TFAEERKEQYKINTAGCKTNEAFYTDILKNKDFNAWSKEYARGFAKTGKSIYYSHASMSH   240
Vf      TFAEERKEQYKINTAGCKTNEAFYTDILKNKDFNAWSKEYARGFAKTGKSIYYSHASMSH   240
        ********************* **:***********************************
```

```
                                                              
F        SWDDWDYAAKVTLANSQKGTAGYIYRFLHDVSEGNDPSVGKNVKELVAYISTSGEKDAGT   300
I        SWDDWDYAAKVTLANSQKGTAGYIYRFLHDVSEGNDPSVGKNVKELVAYISTSGEKDAGT   300
VIII     SWDDWDYAAKVTLANSQKGTAGYIYRFLHDVSEGNDPSVGKNVKELVAYISTSGEKDAGT   300
IVd      SWDDWDYAAKVTLANSQKGTAGYIYRFLHDVSEGNDPSVGKNVKELVAYISTSGEKDAGT   300
IVc      SWDDWDYAAKVTLANSQKGTAGYIYRFLHDVSEGNDPSVGKNVKELVAYISTSGEKDAGT   300
IVb      SWDDWDYAAKVTLANSQKGTAGYIYRFLHDVSEGNDPSVGKNVKELVAYISTSGEKDAGT   300
Vg       SWDYWDYAAKVTLANSQKGTAGYIYRFLHDVSEGNDPSVGKNVKELVAYISTSGEKDAGT   300
Vd       SWDDWDYAAKVTLANSQKGTAGYIYRFLHDVSEGNDPSVGKNVKELVAYISTSGEKDAGT   300
Ve       SWDDWDYAAKVTLANSQKGTAGYIYRFLHDVSEGNDPSVGKNVKELVAYISTSGEKDAGT   300
V        SWDDWDYAAKVTLANSQKGTAGYIYRFLHDVSEGNDPSVGKNVKELVAYISTSGEKDAGT   300
Vf       SWDDWDYAAKVTLANSQKGTAGYIYRFLHDVSEGNDPSVGKNVKELVAYISTSGEKDAGT   300
         *** ********************************************************

           #                                 #                       #
F        DDYMYFGIKTKDGKTQEWEMDNPGNDFMTGSKDTYTFKLKDENLKIDDIQNMWIRKRKYT   360
I        DDYMYFGIKTKDGKTQEWEMDNPGNDFMTGSKDTYTFKLKDENLKIDDIQNMWIRKRKYT   360
VIII     DDYMYFGIKTKDGKTQEWEMDNPGNDFMTGSKDTYTFKLKDENLKIDDIQNMWIRKRKYT   360
IVd      DDYMYFGIKTKDGKTQEWEMDNPGNDFMTGSKDTYTFKLKDENLKIDDIQNMWIRKRKYT   360
IVc      DDYMYFGIKTKDGKTQEWEMDNPGNDFMTGSKDTYTFKLKDENLKIDDIQNMWIRKRKYT   360
IVb      DDYMYFGIKTKDGKTQEWEMDNPGNDFMTGSKDTYTFKLKDENLKIDDIQNMWIRKRKYT   360
Vg       DDYMYFGIKTKDGKTQEWEMDNPGNDFMTGSKDTYTFKLKDENLKIDDIQNMWIRKRKYT   360
Vd       DDYMYFGIKTKDGKTQEWEMDNPGNDFMTGSKDTYTFKLKDENLKIDDIQNMWIRKRKYT   360
Ve       DDYMYFGIKTKDGKTQEWEMDNPGNDFMTGSKDTYTFKLKDENLKIDDIQNMWIRKRKYT   360
V        DDYMYFGIKTKDGKTQEWEMDNPGNDFMTGSKDTYTFKLKDENLKIDDIQNMWIRKRKYT   360
Vf       DDYMYFGIKTKDGKTQEWEMDNPGNDFMTGSKDTYTFKLKDENLKIDDIQNMWIRKRKYT   360
         ************************************************************

          #
F        AFPDAYKPENIKIIANGKVVVDKDINEWISGNSTYNIK       398
I        AFPDAYKPENIKIIANGKVVVDKDINEWISGNSTYNIK       398
VIII     AFPDAYKPENIKVIANGKVVVDKDINEWISGNSTYNIK       398
IVd      AFPDAYKPENIKIIANGKVVVDKDINEWISGNSTYNIK       398
IVc      AFPDAYKPENIKIIANGKVVVDKDINEWISGNSTYNIK       398
IVb      AFPDAYKPENIKIIANGKVVVDKDINEWISGNSTYNIK       398
Vg       AISDAYKPENIKIIANGKVVVDKDINEWISGNSTYNIK       398
Vd       AFPDVYKPENIKVIANGKVVVDKDINEWISGNSTYNIK       398
Ve       AFPDVYKPENIKVIANGKVVVDKDINEWISGNSTYNIK       398
V        AFPDAYKPENIKIIANGKVVVDKDINEWISGNSTYNIK       398
Vf       AFSDAYKPENIKIIANGKVVVDKDINEWISGNSTYNIK       398
         *: *.*******:*************************
```
