# Supplementary material for: Prevalence and Genetic Diversity of Toxin Genes in Clinical Isolates of Clostridium perfringens: Coexistence of Alpha-Toxin Variant and Binary Enterotoxin Genes (bec/cpile)
Source: Toxins (Basel). 2019 Jun 6;11(6):326. doi: 10.3390/toxins11060326 (PMC6628447; doi:10.3390/toxins11060326)
Supplement: Supplementary file 1 [file toxins-11-00326-s001.zip › toxins-514923-si/R1-Figure S2-plc variant.pdf]

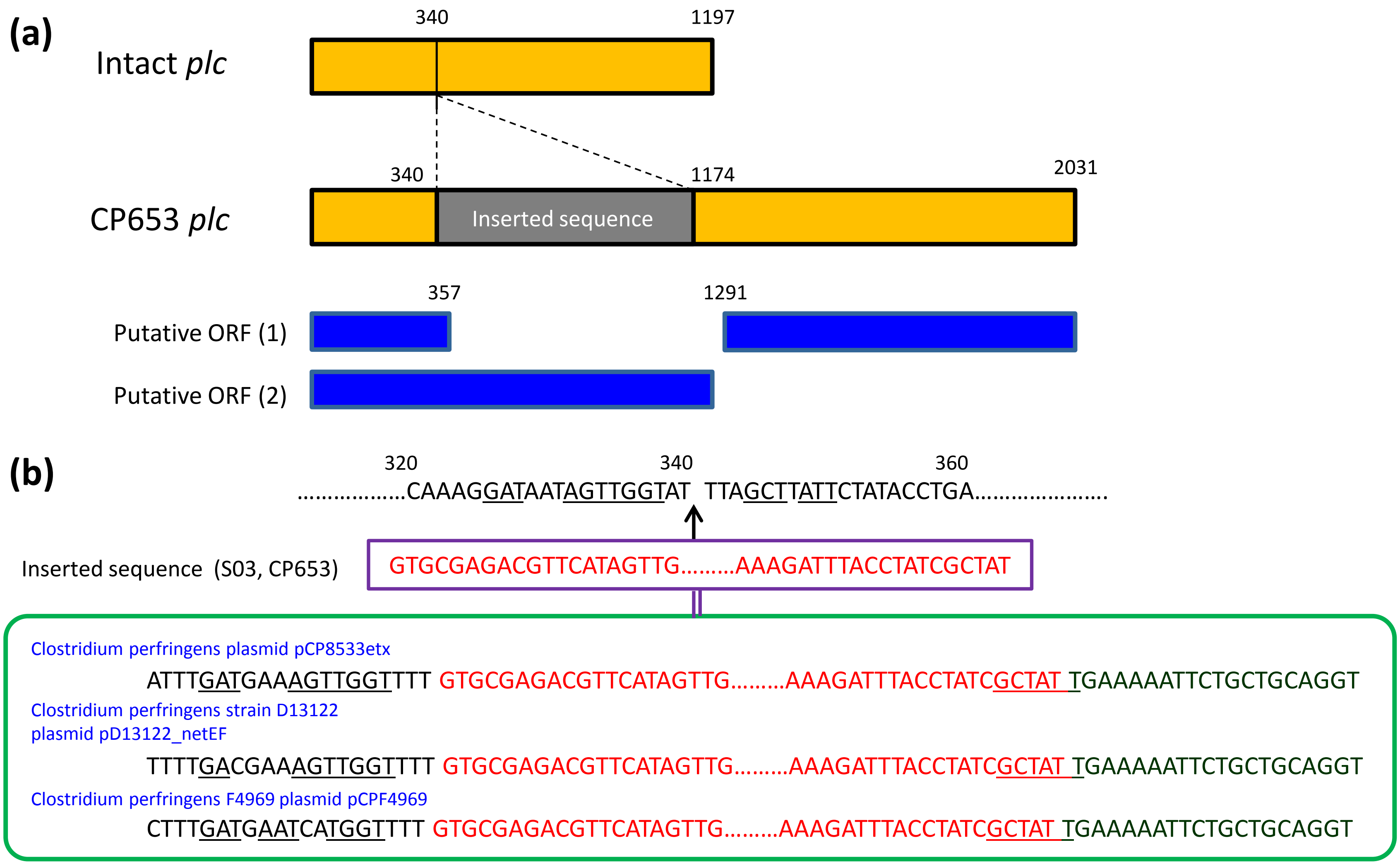

Figure S2 Genetic structure of *plc* variant. (a) Schematic representation of *plc* identified in strain CP653. 834-bp sequence inserted in intact *plc* is shown in gray region. Blue boxes represent putative ORFs (1) deduced by the rearranged nucleotide sequence with the insertion, and another ORF (2) assuming that the inserted sequence is spliced during transcription process.

(b) Position of *plc* sequence where the 834-bp sequence is inserted. Both end parts of the inserted sequence is boxed. Partial *C. perfringens* plasmid sequences containing those in red (both ends) with high similarity to the insertion in CP653 *plc* are shown in a square with green line. Putative consensus sequences around the inserted position in *plc* and the plasmids are underlined.
