# Supplementary material for: Prevalence and Genetic Diversity of Toxin Genes in Clinical Isolates of Clostridium perfringens: Coexistence of Alpha-Toxin Variant and Binary Enterotoxin Genes (bec/cpile)
Source: Toxins (Basel). 2019 Jun 6;11(6):326. doi: 10.3390/toxins11060326 (PMC6628447; doi:10.3390/toxins11060326)
Supplement: Supplementary file 1 [file toxins-11-00326-s001.zip › toxins-514923-si/R1-Figure S3-ab-plc-align.pdf]

**Figure S3  Alignment 834-bp insertion sequence and amino acid sequence of alpha-toxin variant**

**(a)** Alignment of 834-bp insertion sequences in *plc* identified in strains S03 [16], CPBC16ML [17], and CP653 in the present study. Asterisk indicates identical nucleotide.

```
Ins-S03          GTGCGAGACGTTCATAGTTGAAAAGACTATGCACATTCTAAATATAAGAGAATGGAGAAC    60
Ins-CP653        GTGCGAGACGTTCATAGTTGAAAAGACTATGCACATTCTAAATATAAGAGAATGGAGAAC    60
Ins-CPBC16ML     GTGCGAGACGTTCATAGTTGAAAAGACTATGCACATTCTAAATATAAGAGAATGGAGAAC    60
                 ************************************************************

Ins-S03          TCTTAACTTGAAAAGTGGAATGATAGGGTAACGCCTTGAAACGCTTTCTCTAATACTCCG    120
Ins-CP653        TCTTAACTTGAAAAGTGGAATGATAGGGTAACGCCTTGAAACGCTTTCTCTAATACTCCG    120
Ins-CPBC16ML     TCTTAACTTGAAAAGTGGAATGATAGGGTAACGCCTTGAAACGCTTTCTCTAATACTCCG    120
                 ************************************************************

Ins-S03          ACATGCAGTAACTATGTGTGAAAGTTATTGTGTGAAGCTCGGTGAAGTCGGCAGAAAATT    180
Ins-CP653        ACATGCAGTAACTATGTGTGAAAGTTATTGTGTGAAGCTCGGTGAAGTCGGCAGAAAATT    180
Ins-CPBC16ML     ACATGCAGTAACTATGTGTGAAAGTTATTGTGTGAAGCTCGGTGAAGTCGGCAGAAAATT    180
                 ************************************************************

Ins-S03          ACCGTAATAGATAATATTATCTAACGAAAGTGTTCTAGAGGTAGAATATGTAGTTGATAT    240
Ins-CP653        ACCGTAATAGATAATATTATCTAACGAAAGTGTTCTAGAGGTAGAATATGTAGTTGATAT    240
Ins-CPBC16ML     ACCGTAATAGATAATATTATCTAACGAAAGTGTTCTAGAGGTAGAATATGTAGTTGATAT    240
                 ************************************************************

Ins-S03          GCCGGGAGTCGTTAAAAACAGAATATGGTGAGAATGTTCCAATAGGAACTGACGAACTGG    300
Ins-CP653        GCCGGGAGTCGTTAAAAACAGAATATGGTGAGAATGTTCCAATAGGAACTGACGAACTGG    300
Ins-CPBC16ML     GCCGGGAGTCGTTAAAAACAGAATATGGTGAGAATGTTCCAATAGGAACTGACGAACTGG    300
                 ************************************************************

Ins-S03          CGAAGGTACGCTCCTAAGCTATAAATATAGAAATATATTAATAAATTGGTTTGAGGATTA    360
Ins-CP653        CGAAGGTACGCTCCTAAGCTATAAATATAGAAATATATTAATAAATTGGTTTGAGGATTA    360
Ins-CPBC16ML     CGAAGGTACGCTCCTAAGCTATAAATATAGAAATATATTAATAAATTGGTTTGAGGATTA    360
                 ************************************************************

Ins-S03          GTAAGAATAGTACCCATGAAAATCCTTGATATGTTATAGGCATATAATAAGCCAATGGGG    420
Ins-CP653        GTAAGAATAGTACCCATGAAAATCCTTGATATGTTATAGGCATATAATAAGCCAATGGGG    420
Ins-CPBC16ML     GTAAGAATAGTACCCATGAAAATCCTTGATATGTTATAGGCATATAATAAGCCAATGGGG    420
                 ************************************************************

Ins-S03          TAAAGTCAGCACCTAAGTTCTAGTATTGATAAAGTTAAGGGAACGTCGAAAGCTAAAGAC    480
Ins-CP653        TAAAGTCAGCACCTAAGTTCTAGTATTGATAAAGTTAAGGGAACGTCGAAAGCTAAAGAC    480
Ins-CPBC16ML     TAAAGTCAGCACCTAAGTTCTAGTATTGATAAAGTTAAGGGAACGTCGAAAGCTAAAGAC    480
                 ************************************************************

Ins-S03          GTGGAGGATACACACCTATGAAGCGAAGAAAGGGAATACATAACCTTTCATAATCTTTAG    540
Ins-CP653        GTGGAGGATACACACCTATGAAGCGAAGAAAGGGAATACATAACCTTTCATAATCTTTAG    540
Ins-CPBC16ML     GTGGAGGATACACACCTATGAAGCGAAGAAAGGGAATACATAACCTTTCATAATCTTTAG    540
                 ************************************************************

Ins-S03          TGAGAGTAGTGGCATGACTGATGAAGATTTTGTAATGAAATTGGAGGAATAGCCACAAGT    600
Ins-CP653        TGAGAGTAGTGGCATGACTGATGAAGATTTTGTAATGAAATTGGAGGAATAGCCACAAGT    600
Ins-CPBC16ML     TGAGAGTAGTGGCATGACTGATGAAGATTTTGTAATGAAATTGGAGGAATAGCCACAAGT    600
                 ************************************************************

Ins-S03          CAATTATTTATTAAAAGAAACTTAATCAATTCATGGATTCTAGTAAGATAAAAAAGGTCA    660
Ins-CP653        CAATTATTTATTAAAAGAAACTTAATCAATTCATGGATTCTAGTAAGATAAAAAAGGTCA    660
Ins-CPBC16ML     CAATTATTTATTAAAAGAAACTTAATCAATTCATGGATTCTAGTAAGATAAAAAAGGTCA    660
                 ************************************************************

Ins-S03          CTGAATATAGAAAATTAGTGATAGACCATGGCAAGATATTTATATTAATACTTGAGATTA    720
Ins-CP653        CTGAATATAGAAAATTAGTGATAGACCATGGCAAGATATTTATATTAATACTTGAGATTA    720
Ins-CPBC16ML     CTGAATATAGAAAATTAGTGATAGACCATGGCAAGATATTTATATTAATACTTGAGATTA    720
                 ************************************************************
```

```
Ins-S03          AGAAAAATGAATTTAATATATACTTGATGGAACGCCGTATGCGGTGAAAGTCGCACGTAC     780
Ins-CP653        AGAAAAATGAATTTAATATATACTTGATGGAACGCCGTATGCGGTGAAAGTCGCACGTAC     780
Ins-CPBC16ML     AGAAAAATGAATTTAATATATACTTGATGGAACGCCGTATGCGGTGAAAGTCGCACGTAC     780
                 ************************************************************

Ins-S03          GGTGTGGAGCGGGGGAAAAATTGGAGATAACATCAAAGATTTACCTATCGCTAT 834
Ins-CP653        GGTGTGGAGCGGGGGAAAAATTGGAGATAACATCAAAGATTTACCTATCGCTAT 834
Ins-CPBC16ML     GGTGTGGAGCGGGGGAAAAATTGGAGATAACATCAAAGATTTACCTATCGCTAT 834
                 ******************************************************
```

**(b)** Alignment of deduced, spliced amino acid sequences of alpha-toxin in strains S03 and CP653. These sequences are translated through splicing from the original *plc* gene of strains S03 and CP653 (S03-spl, CP653-spl, respectively). Asterisk indicates identical amino acid. Different amino acids are shown in red.

```
                        Signal peptide
                 ________________________
S03-spl          MKRKICKALICAALATSLWAGASTKVYAWDGKIDGTGTHAMIVTQGVSILENDLSKNEPE        60
CP653-spl        MKRKICKALICAALATSLWAGASTKVYAWDGKIDGTGTHAMIVTQGVSILENDLSKNEPE        60
                 ************************************************************

S03-spl          SVRKNLEILKENMHELQLGSTYPDYDKNAYDLYQDHFWDPDTDNNFSKDNSWYLAYSIPD        120
CP653-spl        SVRKNLEILKENMHELQLGSTYPDYDKNAYDLYQDHFWDPDTDNNFSKDNSWYLAYSIPD        120
                 ************************************************************

S03-spl          TGESQIRKFSALARYEWQRGNYKQATFYLGEAMHYFGDIDTPYHPANVTAVDSAGHVKFE        180
CP653-spl        TGESQIRKFSALARYEWQRGNYKQATFYLGEAMHYFGDIDTPYHPANVTAVDSAGHVKFE        180
                 ************************************************************

S03-spl          TFAEERKEQYKINTAGCKTNEAFYTDILKNKDFNAWSKEYARGFAKTGKSIYYSHASMSH        240
CP653-spl        TFAEERKEQYKINTAGCKTNEAFYTDILKNKDFNAWSKEYARGFAKTGKSIYYSHASMSH        240
                 ************************************************************

S03-spl          SWDDWDYAAKVTLANSQKGTAGYIYRFLHDVSEGNDPSVGKNVKELVAYISTSGEKDAGT        300
CP653-spl        SWDYWDYAAKVTLANSQKGTAGYIYRFLHDVSEGNDPSVGKNVKELVAYISTSGEKDAGT        300
                 *** ********************************************************

S03-spl          DDYMYFGIKTKDGKTQEWEMDNPGNDFMTGSKDTYTFKLKDENLKIDDIQNMWIRKRKYT        360
CP653-spl        DDYMYFGIKTKDGKTQEWEMDNPGNDFMTGSKDTYTFKLKDENLKIDDIQNMWIRKRKYT        360
                 ************************************************************

S03-spl          AFSDAYKPENIKIIANGKVVVDKDINEWISGNSTYNIK  398
CP653-spl        AISDAYKPENIKIIANGKVVVDKDINEWISGNSTYNIK  398
                 * ************************************
```
