# Supplementary material for: Prevalence and Genetic Diversity of Toxin Genes in Clinical Isolates of Clostridium perfringens: Coexistence of Alpha-Toxin Variant and Binary Enterotoxin Genes (bec/cpile)
Source: Toxins (Basel). 2019 Jun 6;11(6):326. doi: 10.3390/toxins11060326 (PMC6628447; doi:10.3390/toxins11060326)
Supplement: Supplementary file 1 [file toxins-11-00326-s001.zip › toxins-514923-si/R1-Figure S4-a-cpe.pdf]

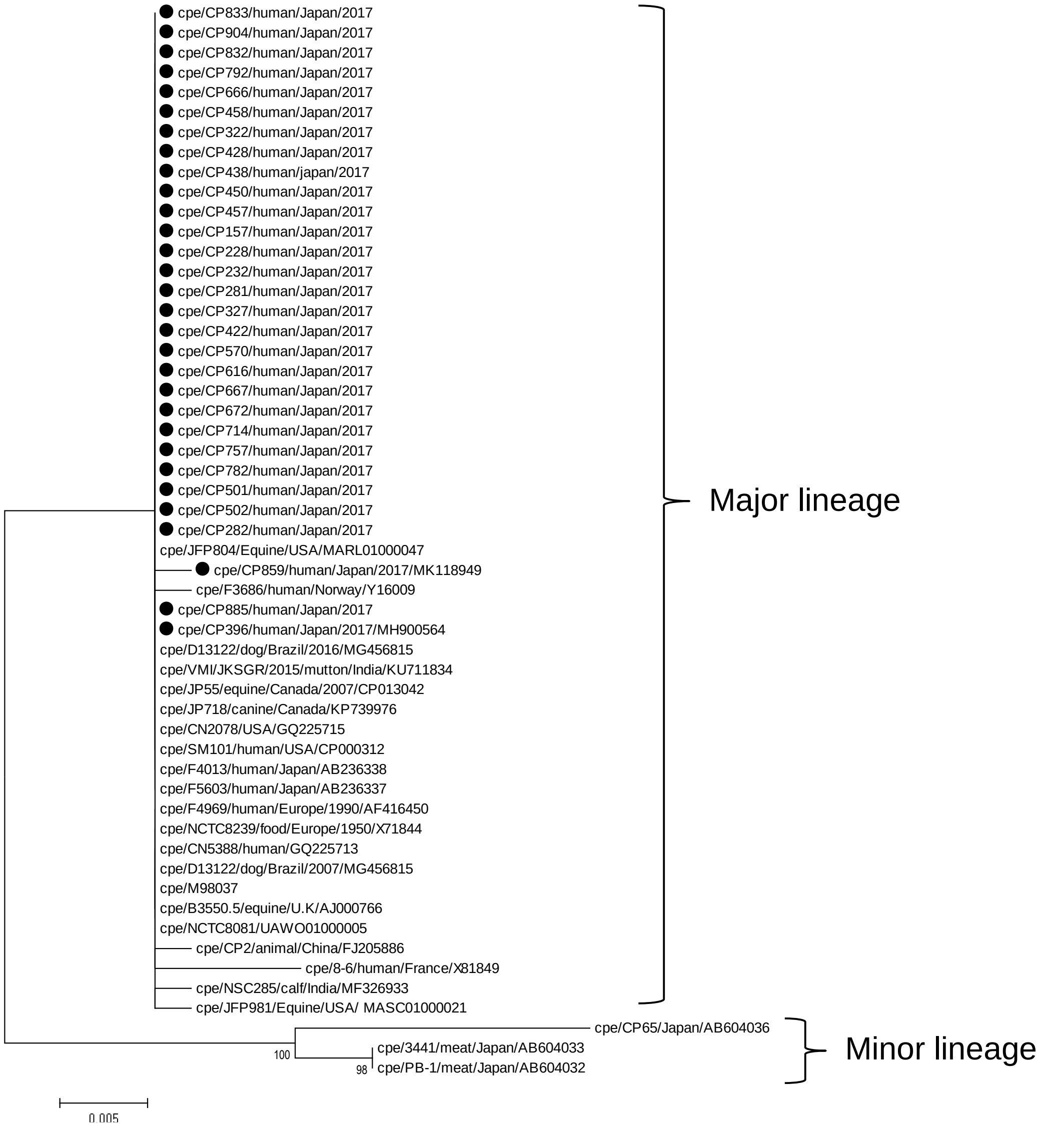

Figure S4. Phylogenetic dendrogram of *cpe* constructed by maximum likelihood method using MEGA7. Tree was statistically supported by bootstrapping with 1000 replicates, and genetic distances were calculated by Kimura two-parameter model. Variation scale is provided at bottom. Percentage bootstrap support is indicated by values at each node (values <80 are omitted). Lineages are shown on the right.
