# Supplementary material for: Prevalence and Genetic Diversity of Toxin Genes in Clinical Isolates of Clostridium perfringens: Coexistence of Alpha-Toxin Variant and Binary Enterotoxin Genes (bec/cpile)
Source: Toxins (Basel). 2019 Jun 6;11(6):326. doi: 10.3390/toxins11060326 (PMC6628447; doi:10.3390/toxins11060326)
Supplement: Supplementary file 1 [file toxins-11-00326-s001.zip › toxins-514923-si/R1-Figure S5-CPE align.pdf]

Figure S5 Alignment of nucleotide and amino acid sequences of CPE

(a) Alignment of *cpe* nucleotide sequences of strains CP396 and CP859. Asterisk indicates identical nucleotide. Different nucleotides are shown in red.

```
CP396          ATGCTTAGTAACAATTTAAATCCAATGGTGTTCGAAAATGCTAAAGAAGTATTTCTTATT 60
CP859          ATGCTTAGTAACAATTTAAATCCAATGGTGTTCGAAAATGCTAAAGAAGTATTTCTTATT 60
               ************************************************************

CP396          TCTGAGGATTTAAAAACACCAATTAATATTACAAACTCTAACTCAAATTTAAGTGATGGA 120
CP859          TCTGAGGATTTAAAAACACCAATTAATATTACAAACTCTAACTCAAATTTAAGTGATGGA 120
               ************************************************************

CP396          TTATATGTAATAGATAAAGGAGATGGTTGGATATTAGGGGAACCCTCAGTAGTTTCAAGT 180
CP859          TTATATGTAATAGATAAAGGAGATGGTTGGATATTAGGGGAACCCTCAGTAGTTTCAAGT 180
               ************************************************************

CP396          CAAATTCTTAATCCTAATGAAACAGGTACCTTTAGCCAATCATTAACTAAATCTAAAGAA 240
CP859          CAAATTCTTAATCCTAATGAAACAGGTACCTTTAGCCAATCATTAACTAAATCTAAAGAA 240
               ************************************************************

CP396          GTATCTATAAATGTAAATTTTTCAGTTGGATTTACTTCTGAATTTATACAAGCATCTGTA 300
CP859          GTATCTATAAATGTAAATTTTTCAGTTGGATTTACTTCTGAATTTATACAAGCATCTGTA 300
               ************************************************************

CP396          GAATATGGATTTGGAATAACTATAGGAGAACAAAATACAATAGAAAGATCTGTATCTACA 360
CP859          GAATATGGATTTGGAATAACTATAGGAGAACAAAATACAATAGAAAGATCTGTATCTACA 360
               ************************************************************

CP396          ACTGCTGGTCCAAATGAATATGTATATTATAAGGTTTATGCAACTTATAGAAAGTATCAA 420
CP859          ACTGCTGGTCCAAATGAATATGTATATTATAAGGTTTATGCAACTTATAGAAAGTATCAA 420
               ************************************************************

CP396          GCTATTAGAATTTCTCATGGTAATATCTCTGATGATGGATCAATTTATAAATTAACAGGA 480
CP859          GCTATTAGAATTTCTCATGGTAATATCTCTGATGATGGATCAATTTATAAATTAACAGGA 480
               ************************************************************

CP396          ATATGGCTTAGTAAAACATCTGCAGATAGCTTAGGAAATATTGATCAAGGTTCATTAATT 540
CP859          ATATGGCTTAGTAAAACATCTGCAGATAGCTTAGGAAATATTGATCAAAGTTCATTAATT 540
               ************************************************ ***********

CP396          GAAACTGGTGAAAGATGTGTTTTAACAGTTCCATCTACAGATATAGAAAAAGAAATCCTT 600
CP859          GAAACTGGTGAAAGATGTGTTTTAACAGTTCCATCTACAGATATAGAAAAAGAAATCCTT 600
               ************************************************************

CP396          GATTTAGCTGCTGCTACAGAAAGATTAAATTTAACTGATGCATTAAACTCAAATCCAGCT 660
CP859          GATTTAGCTGCTGCTACAGAAAGATTAAATTTAACTGATGCATTAAACTCAAATCCAGCT 660
               ************************************************************

CP396          GGTAATTTATATGATTGGCGTTCTTCTAACTCATACCCTTGGACTCAAAAGCTTAATTTA 720
CP859          GGTAATTTATATGATTGGCGTTCTTCTAACTCATACCCTTGGACTCAAAAGCTTAATTTA 720
               ************************************************************

CP396          CACTTAACAATTACAGCTACTGGACAAAAAATATAGAATCTTAGCTAGCAAAATTGTTGAT 780
CP859          CACTTAACAATTACAGCTACTGGACAAAAAATATAGAATCTTAGCTAGCAAAATTGTTGAT 780
               ************************************************************

CP396          TTTAATATTTATTCAAATAATTTTAATAATCTAGTGAAATTAGAACAGTCCTTAGGTGAT 840
CP859          TTTAATATTTATTCAAATAATTTTAATAATCTAGTGAAATTAGAACAGTCCTTAGGTGAT 840
               ************************************************************

CP396          GGAGTAAAAGATCATTATGTTGATATAAGCTTAGATGCTGGACAATATGTTCTTGTAATG 900
CP859          GGAGTAAAAGATCATTATGTTGATATAAGCTTAGATGCTGGACAATATGTTCTTGTAATG 900
               ************************************************************

CP396          AAAGCTAATTCATCATATAGTGGAAATTACCCTTATTCAATATTATTTCAAAAATTTTAA 960
CP859          AAAGCTAATTCATCATATAGTGGAAATTACCCTTATTCAATATTATTTCAAAAATTTTAA 960
               ************************************************************
```

Figure S5 (b) Alignment of CPE amino acid sequences of strains CP396 and CP859. Asterisk indicates identical amino acid. Different amino acid is shown in red.

```
CP396           MLSNNLNPMVFENAKEVFLISEDLKTPINITNSNSNLSDGLYVIDKGDGWILGEPSVVSS 60
CP859           MLSNNLNPMVFENAKEVFLISEDLKTPINITNSNSNLSDGLYVIDKGDGWILGEPSVVSS 60
                ************************************************************

CP396           QILNPNETGTFSQSLTKSKEVSINVNFSVGFTSEFIQASVEYGFGITIGEQNTIERSVST 120
CP859           QILNPNETGTFSQSLTKSKEVSINVNFSVGFTSEFIQASVEYGFGITIGEQNTIERSVST 120
                ************************************************************

CP396           TAGPNEYVYYKVYATYRKYQAIRISHGNISDDGSIYKLTGIWLSKTSADSLGNIDQGSLI 180
CP859           TAGPNEYVYYKVYATYRKYQAIRISHGNISDDGSIYKLTGIWLSKTSADSLGNIDQSSLI 180
                ********************************************************.***

CP396           ETGERCVLTVPSTDIEKEILDLAAATERLNLTDALNSNPAGNLYDWRSSNSYPWTQKLNL 240
CP859           ETGERCVLTVPSTDIEKEILDLAAATERLNLTDALNSNPAGNLYDWRSSNSYPWTQKLNL 240
                ************************************************************

CP396           HLTITATGQKYRILASKIVDFNIYSNNFNNLVKLEQSLGDGVKDHYVDISLDAGQYVLVM 300
CP859           HLTITATGQKYRILASKIVDFNIYSNNFNNLVKLEQSLGDGVKDHYVDISLDAGQYVLVM 300
                ************************************************************

CP396           KANSSYSGNYPYSILFQKF 319
CP859           KANSSYSGNYPYSILFQKF 319
                *******************
```

Figure S5 (c) Alignment of CPE amino acid sequences of *C. perfringens* strains belonging to major and minor lineages. Two strains analyzed in the present study (CP859 and CP396) are shown in yellow. Identical amino acids among all the strains are shown by asterisk at the bottom, while amino acids different from those on the top line (strain 8-6) are indicated in purple. Three domains I, II, III are shown above the sequences. Amino acid residues 44-116 marked in blue are essential for large complex formation and cytotoxicity. Underlined portion (amino acid 290-319) that mediates binding to eukaryotic cell receptor is underlined. Amino acids in red (strain 8-6) are involved in binding to claudin-3/-4.

```
                                                                          II
8-6             ------MLSNNLNPMVFENAKEVFLISEDLKTPINITNSNSNLSDGLYVIDKGDGWILGE 54
B3550.5         ------MVSNNLNPMVFENAKEVFLISEDLKTPINITNSNSNLSDGLYVIDKGDGWILGE 54
F3686           ------MLSNNLNPMVFENAKEVFLISEDLKTPINITNSNSNLSDGLYVIDKGDGWILGE 54
CP2             ------MLSNNLNPMVFENAKEVFLISEDLKTPINITNSNSNLSDGLYVIDKGDGWILGE 54
CP859           ------MLSNNLNPMVFENAKEVFLISEDLKTPINITNSNSNLSDGLYVIDKGDGWILGE 54
F4969           ------MLSNNLNPMVFENAKEVFLISEDLKTPINITNSNSNLSDGLYVIDKGDGWILGE 54
F5603           ------MLSNNLNPMVFENAKEVFLISEDLKTPINITNSNSNLSDGLYVIDKGDGWILGE 54
SM101           ------MLSNNLNPMVFENAKEVFLISEDLKTPINITNSNSNLSDGLYVIDKGDGWILGE 54
JP55            ------MLSNNLNPMVFENAKEVFLISEDLKTPINITNSNSNLSDGLYVIDKGDGWILGE 54
VMI/JKSGR/2015  ------MLSNNLNPMVFENAKEVFLISEDLKTPINITNSNSNLSDGLYVIDKGDGWILGE 54
D13122          ------MLSNNLNPMVFENAKEVFLISEDLKTPINITNSNSNLSDGLYVIDKGDGWILGE 54
CP396           ------MLSNNLNPMVFENAKEVFLISEDLKTPINITNSNSNLSDGLYVIDKGDGWILGE 54
3441            MQNTTNMLSNNLNPMVFENAKEVLLVSEDLKTPINITKSNSNLSDGLYVIDKGDGWILGE 60
CP65            MQNTTNMLSNTLNPMVFENAKEVLLVSEDLKTPINIKNSNSNLSDGLYVIDKGDGWILGE 60
PB-1            MQNTTNMLSNNLNPMVFENAKEVLLVSEDLKTPINITKSNSNLSDGLYVIDKGDGWILGE 60
                      *:**.************:*:**********.:**********************

                                              II
                       III
8-6             PSVVSSQILNPNETGTFSQSLTKSKEVSINVNFSVGFTSEFIQASVEYGFGITIGEQNTI 114
B3550.5         PSVVSSQILNPNETGTFSQSLTKSKEVSINVNFSVGFTSEFIQASVEYGFGITIGEQNTI 114
F3686           PSVVSSQILNPNETGTFSQSLTKSKEVSINVNFSVGFTSEFIQASVEYGFGITIGEQNTT 114
CP2             PSVVSSQILNPNETGTFSQSLTKSKEVSINVNFSVGFTSEFIQASVEYGFGITIGEQNTI 114
CP859           PSVVSSQILNPNETGTFSQSLTKSKEVSINVNFSVGFTSEFIQASVEYGFGITIGEQNTI 114
F4969           PSVVSSQILNPNETGTFSQSLTKSKEVSINVNFSVGFTSEFIQASVEYGFGITIGEQNTI 114
F5603           PSVVSSQILNPNETGTFSQSLTKSKEVSINVNFSVGFTSEFIQASVEYGFGITIGEQNTI 114
SM101           PSVVSSQILNPNETGTFSQSLTKSKEVSINVNFSVGFTSEFIQASVEYGFGITIGEQNTI 114
JP55            PSVVSSQILNPNETGTFSQSLTKSKEVSINVNFSVGFTSEFIQASVEYGFGITIGEQNTI 114
VMI/JKSGR/2015  PSVVSSQILNPNETGTFSQSLTKSKEVSINVNFSVGFTSEFIQASVEYGFGITIGEQNTI 114
D13122          PSVVSSQILNPNETGTFSQSLTKSKEVSINVNFSVGFTSEFIQASVEYGFGITIGEQNTI 114
CP396           PSVVSSQILNPNETGTFSQSLTKSKEVSINVNFSVGFTSEFIQASVEYGFGITIGEQNTI 114
3441            PSVVSSQILNPNETGTFSQSLTKSKEVSINVNFSVGFTSEFIQASVEYGFGITIGEQNTI 120
CP65            PSVVSSQILNPNETGTFSQSLTKSKEVSINVNFSVGFTSEFIQASVEYGFGITIGEQNTI 120
PB-1            PSVVSSQILNPNETGTFSQSLTKSKEVSINVNFSVGFTSEFIQASVEYGFGITIGEQNTI 120
                ***********************************************************

                                           II
                       III
8-6             ERSVSTTAGPNEYVVYYKVYATYRKYQAIRISHGNISDSGSIYKLTGIWLMKTSADSLGNI 174
B3550.5         ERSVSTTAGPNEYVVYYKVYATYRKYQAIRISHGNISDDGSIYKLTGIWLSKTSADSLGNI 174
F3686           ERSVSTTAGPNEYVVYYKVYATYRKYQAIRISHGNISDDGSIYKLTGIWLSKTSADSLGNI 174
CP2             ERSVSTTAGPNEYVVYYKVYATYRKYQAIRISHGNISDDGSIYKLTGIWLSKTSADSLGNI 174
CP859           ERSVSTTAGPNEYVVYYKVYATYRKYQAIRISHGNISDDGSIYKLTGIWLSKTSADSLGNI 174
F4969           ERSVSTTAGPNEYVVYYKVYATYRKYQAIRISHGNISDDGSIYKLTGIWLSKTSADSLGNI 174
F5603           ERSVSTTAGPNEYVVYYKVYATYRKYQAIRISHGNISDDGSIYKLTGIWLSKTSADSLGNI 174
SM101           ERSVSTTAGPNEYVVYYKVYATYRKYQAIRISHGNISDDGSIYKLTGIWLSKTSADSLGNI 174
JP55            ERSVSTTAGPNEYVVYYKVYATYRKYQAIRISHGNISDDGSIYKLTGIWLSKTSADSLGNI 174
VMI/JKSGR/2015  ERSVSTTAGPNEYVVYYKVYATYRKYQAIRISHGNISDDGSIYKLTGIWLSKTSADSLGNI 174
D13122          ERSVSTTAGPNEYVVYYKVYATYRKYQAIRISHGNISDDGSIYKLTGIWLSKTSADSLGNI 174
CP396           ERSVSTTAGPNEYVVYYKVYATYRKYQAIRISHGNISDDGSIYKLTGIWLSKTSADSLGNI 174
3441            ERSVSTTAGPNEYVVYYKVYATYRKYQAIRISHGNISDDGSIYKLTGIWLSKTSADSLANI 180
CP65            QRSVSTTAGPNEYVVYYKVYATYRKYQVIRISHGNISDDGSIYKLTGIWLSKTSADSLANI 180
PB-1            ERSVSTTAGPNEYVVYYKVYATYRKYQAIRISHGNISDDGSIYKLTGIWLSKTSADSLANI 180
                :**************************.**********.*********** *******.**
```

```
                    II
         III
8-6            DQGSLIETGERCVLTVPSTDIEKEILDLAAATERLNLTDALNSNPAGNLYDWRSSNSYPW 234
B3550.5        DQGSLIETGERCVLTVPSTDIEKEILDLAAATERLNLTDALNSNPAGNLYDWRSSNSYPW 234
F3686          DQGSLIETGERCVLTVPSTDIEKEILDLAAATERLNLTDALNSNPAGNLYDWRSSNSYPW 234
CP2            DQGSLIETGERCVLTVPSTDIEKEILDLAAATERLNLTDALNSNPAGNLYDWRSSNSYPW 234
CP859          DQSSLIETGERCVLTVPSTDIEKEILDLAAATERLNLTDALNSNPAGNLYDWRSSNSYPW 234
F4969          DQGSLIETGERCVLTVPSTDIEKEILDLAAATERLNLTDALNSNPAGNLYDWRSSNSYPW 234
F5603          DQGSLIETGERCVLTVPSTDIEKEILDLAAATERLNLTDALNSNPAGNLYDWRSSNSYPW 234
SM101          DQGSLIETGERCVLTVPSTDIEKEILDLAAATERLNLTDALNSNPAGNLYDWRSSNSYPW 234
JP55           DQGSLIETGERCVLTVPSTDIEKEILDLAAATERLNLTDALNSNPAGNLYDWRSSNSYPW 234
VMI/JKSGR/2015 DQGSLIETGERCVLTVPSTDIEKEILDLAAATERLNLTDALNSNPAGNLYDWRSSNSYPW 234
D13122         DQGSLIETGERCVLTVPSTDIEKEILDLAAATERLNLTDALNSNPAGNLYDWRSSNSYPW 234
CP396          DQGSLIETGERCVLTVPSTDIEKEILDLAAATERLNLTDALNSNPAGNLYDWRSSNSYPW 234
3441           DQGSLIETGERCVLTVPSSDIEKEILDLAAATERLNLTDALNTNPAGNLYDWRSSNSYPW 240
CP65           DQGSLIETGERCVLTVPPSDIEKEILDLAAATERLNLTDALNTNPAGNLYDWRSSNSYPW 240
PB-1           DQGSLIETGERCVLTVPSSDIEKEILDLAAATERLNLTDALNTNPAGNLYDWRSSNSYPW 240
               **.**************.:***********************:*****************

                                         I
8-6            TQKLNLHLTITATGQKYRILASKIVDFNIYSNNFNNLVKLEQSLGDGVKDHYVDISLDAG 294
B3550.5        TQKLNLHLTITATGQKYRILASKIVDFNIYSNNFNNLVKLEQSLGDGVKDHYVDISLDAG 294
F3686          TQKLNLHLTITATGQKYRILASKIVDFNIYSNNFNNLVKLEQSLGDGVKDHYVDISLDAG 294
CP2            TQKLNLHLTITATGQKYRILASKIVDFNIYSNNFNNLVKLEQSLGDGVKDHYVDISLDAE 294
CP859          TQKLNLHLTITATGQKYRILASKIVDFNIYSNNFNNLVKLEQSLGDGVKDHYVDISLDAG 294
F4969          TQKLNLHLTITATGQKYRILASKIVDFNIYSNNFNNLVKLEQSLGDGVKDHYVDISLDAG 294
F5603          TQKLNLHLTITATGQKYRILASKIVDFNIYSNNFNNLVKLEQSLGDGVKDHYVDISLDAG 294
SM101          TQKLNLHLTITATGQKYRILASKIVDFNIYSNNFNNLVKLEQSLGDGVKDHYVDISLDAG 294
JP55           TQKLNLHLTITATGQKYRILASKIVDFNIYSNNFNNLVKLEQSLGDGVKDHYVDISLDAG 294
VMI/JKSGR/2015 TQKLNLHLTITATGQKYRILASKIVDFNIYSNNFNNLVKLEQSLGDGVKDHYVDISLDAG 294
D13122         TQKLNLHLTITATGQKYRILASKIVDFNIYSNNFNNLVKLEQSLGDGVKDHYVDISLDAG 294
CP396          TQKLNLHLTITATGQKYRILASKIVDFNIYSNNFNNLVKLEQSLGDGVKDHYVDISLDAG 294
3441           TQKLNLHLTITATGQKYRILASRIVDFNIYSNNFNNLVKLQESLGDGVNDHYIDISLDAG 300
CP65           TQKLNLHLTITATGQKYRILASRIVDFNIYSNNFNNLVKLQESLGDGVNDHYIDISLDAG 300
PB-1           TQKLNLHLTITATGQKYRILASRIVDFNIYSNNFNNLVKLQESLGDGVNDHYIDISLDAG 300
               **********************:*****************::******:***:****** 

8-6            QYVLVMKANSSYSGNYPYSILFQKF 319
B3550.5        QYVLVMKANSSYSGNYPYSILFQKF 319
F3686          QYVLVMKANSSYSGNYPYSILFQKF 319
CP2            QYVLVMKANSSYSGNYPYSILFQKF 319
CP859          QYVLVMKANSSYSGNYPYSILFQKF 319
F4969          QYVLVMKANSSYSGNYPYSILFQKF 319
F5603          QYVLVMKANSSYSGNYPYSILFQKF 319
SM101          QYVLVMKANSSYSGNYPYSILFQKF 319
JP55           QYVLVMKANSSYSGNYPYSILFQKF 319
VMI/JKSGR/2015 QYVLVMKANSSYSGNYPYSILFQKF 319
D13122         QYVLVMKANSSYSGNYPYSILFQKF 319
CP396          QYVLVMKANSSYSGNYPYSILFQKF 319
3441           QYVLVMKANSSYSGNYPYAILFQKF 325
CP65           QYVLVMKANSSYSGNY--------- 316
PB-1           QYVLVMKANSSYSGNYPYAILFQKF 325
               ****************
```
