# Supplementary material for: Prevalence and Genetic Diversity of Toxin Genes in Clinical Isolates of Clostridium perfringens: Coexistence of Alpha-Toxin Variant and Binary Enterotoxin Genes (bec/cpile)
Source: Toxins (Basel). 2019 Jun 6;11(6):326. doi: 10.3390/toxins11060326 (PMC6628447; doi:10.3390/toxins11060326)
Supplement: Supplementary file 1 [file toxins-11-00326-s001.zip › toxins-514923-si/R1-Figure S6-cpb2.pdf]

Figure S6 Alignment of beta2 toxin amino acid sequences representing cluster 1 (strain CP301) and cluster 2 (strains JGS1902, JGS1984), and prototype (CWC412) and prototype-related isolate CP412. Asterisk indicates identical amino acid.

```
CP412           MKKIISKVTVIFMFSCFLIVGAISPMKASAKEIDAYRKVMENYLNALKNYDINTVVNISE 60
CWC245          MKKIISKFTVIFMFSCFLIVGAISPMKASAKEIDAYRKVMENYLNALKNYDINTVVNISE 60
CP301           MKKIISKFTVIFMFSCFLIVGAISPMKASAKEIDTYREVMENYLNAFKNYDINTIVNVSE 60
JGS1902         MKKLIVKSTMMLLFSCLLCLGIQLPNTVKANEVNKYQSVMVQYLEAFKNYDIDTIVDISK 60
JGS1984         MKKLIVKSTMMLLFSCLLCLGIQLPNTVKANEVNKYQSVMVQYLEAFKNYDIDTIVDISK 60
                ***:* * *::::***:* :*   * ...*:*:: *:.** :**:*:*****:*:*::*:

CP412           DERVNNVEQYREMLEDFKSDPNQQLKSFEILNSQKRDNKEIFNVKTEFLNGAIYDMEFTV 120
CWC245          DERVNNVEQYREMLEDFKYDPNQQLKSFEILNSQKSDNKEIFNVKTEFLNGAIYDMEFTV 120
CP301           DERVNSDEKYKEMLEEFKYDPNQQLKSFEILNSQKSDNKEIFNVKTEFMNGAIYDMKFTV 120
JGS1902         DSRAVTKEEYKNMLMEFKYDPNQKLKSYEITSSRKIDNSEIFSVKTEFLNGAIYNMEFTV 120
JGS1984         DSRAVTKEEYKNMLMEFKYDPNQKLKSYEITGSRKIDNGEIFSVKTEFLNGAIYNMEFTV 120
                *.*. . *:*::** :** ****:***:** .*:* ** ***.*****:*****:*:***

CP412           SSKDGKLIVSNMERTKIENEGKYILTPSFRTQVCTWDDELGQAIGGVYPQTYSDRFTYYA 180
CWC245          SSKDGKLIVSDMERTKVENEGKYILTPSFRTQVCTWDDELAQAIGGVYPQTYSDRFTYYA 180
CP301           SSKDGKLIVSDMERTKIENEGKYILTPSFRTQVCTWDDELSQSIGGVDPKTYSTRFTYYA 180
JGS1902         SYIDNKLMVSDMNRIAIINEGKRIPTPSFRTQVCTWDDELSQYIGDAVSFTRSSKFQYSS 180
JGS1984         SYIDNKLMVSNMNRISIVNEGKCIPTPSFRTQVCTWDDELSQYIGDAVSFTRSSKFQYSS 180
                *  *.**:**:*:*  : **** * ***************.* **.. . * * :* * :

CP412           DNILLNFRQYATSGSRDLRVEYSVVDHWMWKDDVKASQMVYGQNPDSARQIRLYIEKGQS 240
CWC245          DNILLNFRQYATSGSRDLKVEYSVVDHWMWKDDVKASQMVYGQNPDSARQIRLYIEKGQS 240
CP301           DNILLNFRQYATSGSRDLKVEYSVVDHWVWGDDVKASQMVYGQNPDSARQIRLYIEKGQS 240
JGS1902         NTITLNFRQYATSGSRSLKVKYSVVDHWIWGDDIRASQWVYGENPDYARQIKLHLGSGET 240
JGS1984         NTITLNFRQYATSGSRSLKVKYSVVDHWMWGDDIRASQWVYGENPDYARQIKLYLGSGET 240
                :.* ************.*:*:*******:* **::*** ***:*** ****:*:: .*::

CP412           FYKYRIRIKNFTPASLKVFGEGYCA 265
CWC245          FYKYRIRIKNFTPASIRVFGEGYCA 265
CP301           FYKYRIRIQNFTPASIKVFGEGYCA 265
JGS1902         FQNYRIKVENYTPASIRVFGEGYCY 265
JGS1984         FKNYRIKVENYTPASIKVFGEGYCY 265
                * :***:::*:****::*******
```
