# Supplementary material for: Prevalence and Genetic Diversity of Toxin Genes in Clinical Isolates of Clostridium perfringens: Coexistence of Alpha-Toxin Variant and Binary Enterotoxin Genes (bec/cpile)
Source: Toxins (Basel). 2019 Jun 6;11(6):326. doi: 10.3390/toxins11060326 (PMC6628447; doi:10.3390/toxins11060326)
Supplement: Supplementary file 1 [file toxins-11-00326-s001.zip › toxins-514923-si/R1-Figure S7-BEC-alignment.pdf]

Figure S7 Alignment of BECab/CPILE-ab amino acid sequences

(a) Amino acid sequence alignment of BECa (CPILE-a) (1) and BECb (CPILE-b) (2) of strains OS1, TS1, W5052 (GenBank accession nos. AP013033, AP13034, AB921559, respectively) and CP653 analyzed in the present study. Amino acids in CP653 proteins which are different from those of other three strains are shown in red, while asterisk indicates identical amino acid. A short loop sequence (residue 210-216) linking structurally homologous N-terminal domain and C-terminal domain is represented by shading above the sequence. Positions marked in yellow below the sequences represent common structural traits in ADP-ribosylating toxin (ADPRT)-family proteins, i.e., an aromatic residue (Y)-R/H, S-T-S motif (S-T-T in BECa/CPILE-a), an E-X-E motif in the ADP-ribosylating turn-turn (ARTT) loop underlined. Conserved amino acids associated with NADH recognition [26] are indicated by blue bars above the sequences. Loop I-V, and two protruding loops PT-I and PT-II associated with binding to actin [27] are indicated by green and red boxes, respectively.

(1) BECa / CPILE-a

```
CP653    MLDDNRPMDFAKDKNSATLWAKKRKQVWLNNLSKAESTSIDNYIKNSSEINSYSIKKKFA   60
OS1      MLDDNRPMDFAKDKNSATLWAKKRKQVWLNNLSKAESTSINNYIKNSSEINSYSIKKKFA   60
TS-1     MLDDNRPMDFAKDKNSATLWAKKRKQVWLNNLSKAESTSINNYIKNSSEINSYSIKKKFA   60
W5052    MLDDNRPMDFAKDKNSATLWAKKRKQVWLNNLSKAESTSINNYIKNSSEINSYSIKKKFA   60
         ****************************************:*******************
                           α1                        α2

CP653    LDNYEGIETLNEDLKNISTSVKKSMLTKPLYVYYYETNDKFGFNQNLESSLDSNIIDDEA   120
OS1      LDNYEGIETLNEDLKNISTAVKKSMLTKPLYVYYYEANDKFGFNQNLESSLDSNIIDEEA   120
TS-1     LDNYEGIETLNEDLKNISTAVKKSMLTKPLYVYYYEANDKFGFNQNLESSLDSNIIDEEA   120
W5052    LDNYEGIETLNEDLKNISTAVKKSMLTKPLYVYYYEANDKFGFNQNLESSLDSNIIDEEA   120
         *******************:****************:*********************:**
        loop I          α3                  β1

CP653    INNFAKKISDTNFIQDGFKDVNMTEPDINSKLPILVHLKLPTNTPAASYGNDEENLRVLI   180
OS1      INNFAKKISDTNFIQDGFKDVTMTEPDINSKLPILVHLKLPTNTPAASYGNDEENLRVLI   180
TS-1     INNFAKKISDTNFIQDGFKDVTMTEPDINSKLPILVHLKLPTNTPAASYGNDEENLRVLI   180
W5052    INNFAKKISDTNFIQDGFKDVTMTEPDINSKLPILVHLKLPTNTPAASYGNDEENLRVLI   180
         *********************.**************************************
            α4          β2     β3               β4

                N-terminal domain ←         → C-terminal domain
CP653    DQGYSLKATGLSIVTIKGKQYAKVDADLIKQLNFENDVISASQWGEENYAPWLKELTSNE   240
OS1      DQGYSLKATGLSIVTIKGKQYAKVDADLIKQLNFENDVISASQWGEENYAPWLKELTSNE   240
TS-1     DQGYSLKATGLSIVTIKGKQYAKVDADLIKQLNFENDVISASQWGEENYAPWLKELTSNE   240
W5052    DQGYSLKATGLSIVTIKGKQYAKVDADLIKQLNFENDVISASQWGEENYAPWLKELTSNE   240
         ************************************************************

CP653    LRDINNYLGGGYTAINKYLLDGTIGENTSKEDLEEKISNISSALKKKKIPEDIITYRRMG   300
OS1      LRDINNYLGGGYTAINKYLLDGTIGENTSKEDLEEKISNISSALKKRKIPEDIITYRRMG   300
TS-1     LRDINNYLGGGYTAINKYLLDGTIGENTSKEDLEEKISNISSALKKRKIPEDIITYRRMG   300
W5052    LRDINNYLGGGYTAINKYLLDGTIGENTSKEDLEEKISNISSALKKRKIPEDIITYRRMG   300
         **********************************************:*************
                 loop II         PT-I            Aromatic residue (Y)-R/H

CP653    PNEFGLDLNSPDYDFNKVENVSKFKEKWLGKTIPVKTFISTTVLSNNISAFAKRKLILRL   360
OS1      PNEFGLDLNSPDYDFNKVENVSKFKEKWLGKTIPVKTFISTTVLSNNISAFAKRKLILRL   360
TS-1     PNEFGLDLNSPDYDFNKVENVSKFKEKWLGKTIPVKTFISTTVLSNNISAFAKRKLILRL   360
W5052    PNEFGLDLNSPDYDFNKVENVSKFKEKWLGKTIPVKTFISTTVLSNNISAFAKRKLILRL   360
         ************************************************************
              loop III                          S-T-S motif     loop IV

CP653    HLPTGSNAAYVSVAEGYKNEYEVLIDHGYSYKIDNITEYYDESSLGGKTNKLIIDATLI   419
OS1      HLPNGSNAAYVSVAEGYKNEYEVLIDHGYSYKIDNITEYYDESSLGGKTNKLIIDATLI   419
TS-1     HLPNGSNAAYVSVAEGYKNEYEVLIDHGYSYKIDNITEYYDESSLGGKTNKLIIDATLI   419
W5052    HLPNGSNAAYVSVAEGYKNEYEVLIDHGYSYKIDNITEYYDESSLGGKTNKLIIDATLI   419
         ***.*******************************************************
             ARTT loop (E-X-E motif) / loop V          PT-II
```

(2) BECb / CPILE-b

```
CP653     MINNTFFMGYYFSDSDFDNLLFMSPTEGNILSYSKDIVEDLFNKNSTFIQSIRWLGKIKV    60
TS1       MINNTFFMGYYFSDSDFDNLLFMSPTEGNILSYSKDIVEDLFNKNSTFIQSIRWLGKIKV    60
OS1       MINNTFFMGYYFSDSDFDNLLFMSPTEGNILSYSKDIVEDLFNKNSTFIQSIRWLGKIKV    60
W5052     MINNTFFMGYYFSDSDFDNLLFMSPTEGNILSYSKDIVEDLFNKNSTFIQSIRWLGKIKV    60
          ************************************************************

CP653     DIDGVYEFLINSDTLIELDKTLLNSTSNKLKLKKDIFYNIRIEQKLENKTLLKDFVPKMF    120
TS1       DIDGIYEFLINPDTLIELDKTILNSTSNKLKLKKDIFYNIRIEQKLENKTLLKDFVPKMF    120
OS1       DIDGIYEFLINPDTLIELDKTILNSTSNKLKLKKDIFYNIRIEQKLENKTLLKDFVPKMF    120
W5052     DIDGIYEFLINPDTLIELDKTILNSTSNKLKLKKDIFYNIRIEQKLENKTLLKDFVPKMF    120
          ****:****** *********:**************************************

CP653     QINLLNNSNFKTSNKHQIYSPNFSKTDEITFIPNESLFGSKLKIEVKKDTDNDNIQDDWE    180
TS1       QINLLSNSNFKTSNKHQIYSPNFSKTDEITFIPNESLFGSKLKIEVKKDTDNDNIQDDWE    180
OS1       QINLLSNSNFKTSNKHQIYSPNFSKTDEITFIPNESLFGSKLKIEVKKDTDNDNIQDDWE    180
W5052     QINLLSNSNFKTSNKHQIYSPNFSKTDEITFIPNESLFGSKLKIEVKKDTDNDNIQDDWE    180
          *****.******************************************************

CP653     LNGYTIKDKVAAKWQDEYANLGYKKFVSNPFNSHTANDPYTDYEKASMDYDQSILQVAQD    240
TS1       LNGYTIKDKVAAKWKDEYANLGYKKFVSNPFNSHTANDPYTDYEKAAMDYDQSILQVAQD    240
OS1       LNGYTIKDKVAAKWKDEYANLGYKKFVSNPFNSHTANDPYTDYEKAAMDYDQSILQVAQD    240
W5052     LNGYTIKDKVAAKWKDEYANLGYKKFVSNPFNSHTANDPYTDYEKAAMDYDQSILQVAQD    240
          **************:*******************************:*************

CP653     PMVAAYPVVGVKMEKLILSKNQISSTEQGKTISRATSNSSTSSITSSISTNIGFNPEGIS    300
TS1       PMVAAYPVVGVKMEKLILSKNQISSTEQGKTISRATSNSSTSSITSSISTNIGFNPEGIS    300
OS1       PMVAAYPVVGVKMEKLILSKNQISSTEQGKTISRATSNSSTSSITSSISTNIGFNPEGIS    300
W5052     PMVAAYPVVGVKMEKLILSKNQISSTEQGKTISRATSNSSTSSITSSISTNIGFNPEGIS    300
          ************************************************************

CP653     GGVTTEFSNTHETSNTVTNETGETWSEGLQINIGESAYLNANIRYYNSGSAPIYDVQPTT    360
TS1       GGVTTEFSNTHETSNTVTNETGETWSEGLQINIGESAYLNANIRYYNSGSAPIYDVQPTT    360
OS1       GGVTTEFSNTHETSNTVTNETGETWSEGLQINIGESAYLNANIRYYNSGSAPIYDVQPTT    360
W5052     GGVTTEFSNTHETSNTVTNETGETWSEGLQINIGESAYLNANIRYYNSGSAPIYDVQPTT    360
          ************************************************************

CP653     NLVLDNDTINTILAGPNQLANVLQADKVYPAPNLNPIAMNTMDPSNSLFIHVNYDQLKKL    420
TS1       NLVLDNDTINTILAGPNQLANVLQADKVYPAPNLNPIAMNTMDPSNSLFIHVNYDQLKKL    420
OS1       NLVLDNDTINTILAGPNQLANVLQADKVYPAPNLNPIAMNTMDPSNSLFIHVNYDQLKKL    420
W5052     NLVLDNDTINTILAGPNQLANVLQADKVYPAPNLNPIAMNTMDPSNSLFIHVNYDQLKKL    420
          ************************************************************

CP653     DNGIKLSLETTQVKGNFLTRNNQGESSTEGNDWKNYMSQIENTCTLLILDNKGEVFERYV    480
TS1       DNGIKLSLETTQVKGNFLTRNNQGESTTEGNDWKNYMSQIENTCALLILDNKGEVFERYV    480
OS1       DNGIKLSLETTQVKGNFLTRNNQGESTTEGNDWKNYMSQIENTCALLILDNKGEVFERYV    480
W5052     DNGIKLSLETTQVKGNFLTRNNQGESTTEGNDWKNYMSQIENTCALLILDNKGEVFERYV    480
          **************************:*****************:***************

CP653     AAPNNSNPEDKTPQLTLREALIKAFSLEERENKLYYKDIPFNEDDILLVYDELTKNEIEK    540
TS1       AAPNSSNPEDKTPQLTLREALIKAFSLEERENKLYYKDIPFNEDDILLVYDELTKNEIEK    540
OS1       AAPNSSNPEDKTPQLTLREALIKAFSLEERENKLYYKDIPFNEDDILLVYDELTKNEIEK    540
W5052     AAPNSSNPEDKTPQLTLREALIKAFSLEERENKLYYKDIPFNEDDILLVYDELTKNEIEK    540
          ****.*******************************************************

CP653     QIASLDKKEIYNIKINPKMKILAKIPVYICDFDKYNKVWANVNIIDDPSRGKVACISKDT    600
TS1       QIAGLDKKEIYNIKINPKMKILAKIPVYICDFDKYNKVWANVNIIDDPSRGKVACISKNT    600
OS1       QIAGLDKKEIYNIKINPKMKILAKIPVYICDFDKYNKVWANVNIIDDPSRGKVACISKNT    600
W5052     QIAGLDKKEIYNIKINPKMKILAKIPVYICDFDKYNKVWANVNIIDDPSRGKVACISKNT    600
          ***.******************************************************:*

CP653     AVANTIISTLEPKKSYMLNMYIKGQNKNKVSFAVQSEKPLEVELTENYRQISIPFKTLIG    660
TS1       TVANTIISTLEPKKSYMLNMYIKGQNKNKVSFAVQSEKPLEVELTENYRQISIPFKTLIG    660
OS1       TVANTIISTLEPKKSYMLNMYIKGQNKNKVSFAVQSEKPLEVELTENYRQISIPFKTLIG    660
W5052     TVANTIISTLEPKKSYMLNMYIKGQNKNKVSFAVQSEKPLEVELTENYRQISIPFKTLIG    660
          :***********************************************************

CP653     KDVYININNLSKNGDIYFDNVIITEIDEFDPSKEDIKKKNGDIYSFYKDNSSYLYRCDFN    720
TS1       KDVYININNLSKNGDIYFDNVIITEIDEFDPSREDIKKKNGDIYSFYKDNSSYLYRCDFN    720
OS1       KDVYININNLSKNGDIYFDNVIITEIDEFDPSREDIKKKNGDIYSFYKDNSSYLYRCDFN    720
W5052     KDVYININNLSKNGDIYFDNVIITEIDEFDPSREDIKKKNGDIYSFYKDNSSYLYRCDFN    720
          ********************************:***************************
```

```
CP653      IPYLKYFPKIGLRAFVNGIYKDLGLRFKNHDGHKTILTPTDYTDSPVKIDQFEVYVIDQY     780
TS1        IPFLKYFPQIGLRAFVNGIYKDLGLRLRNHDGYKTIITPADYTNSPVQIDQFEVYVIDQY     780
OS1        IPFLKYFPQIGLRAFVNGIYKDLGLRLRNHDGYKTIITPADYTNSPVQIDQFEVYVIDQY     780
W5052      IPFLKYFPQIGLRAFVNGIYKDLGLRLRNHDGYKTIITPADYTNSPVQIDQFEVYVIDQY     780
           **:*****:*****************::****:***:**:***:***:************

CP653      GDPLTLFTSKDTIVLKYPF    799
TS1        GDPLTLFTSKDTIVLEYPF    799
OS1        GDPLTLFTSKDTIVLEYPF    799
W5052      GDPLTLFTSKDTIVLEYPF    799
           ***************:***
```
