# Supplementary material for: Prevalence and Genetic Diversity of Toxin Genes in Clinical Isolates of Clostridium perfringens: Coexistence of Alpha-Toxin Variant and Binary Enterotoxin Genes (bec/cpile)
Source: Toxins (Basel). 2019 Jun 6;11(6):326. doi: 10.3390/toxins11060326 (PMC6628447; doi:10.3390/toxins11060326)
Supplement: Supplementary file 1 [file toxins-11-00326-s001.zip › toxins-514923-si/R1-TableS1234.pdf]

Table S1 Alpha-toxin (phospholipase C) sequence types assigned to 46 *C. perfringens* isolates

Strain ID	amino acid position of alpha-toxin											Sequence type
	13	54	57	166	202	205	244	362	363	365	373	
Strain 13	T	L	N	A	D	A	D	F	P	A	I	Type F*
CP201	A	L	N	A	D	A	D	F	P	A	I	Type I
CP238	A	L	N	A	D	A	D	F	P	A	I	Type I
CP606	A	L	N	A	D	A	D	F	P	A	I	Type I
CP501	A	M	N	A	D	A	D	F	P	A	I	Type IVb
CP322	A	M	N	A	D	A	D	F	P	A	I	Type IVb
CP282	A	M	N	A	D	A	D	F	P	A	I	Type IVb
CP904	A	M	N	A	D	A	D	F	P	A	I	Type IVb
CP648	A	M	N	A	D	A	D	F	P	A	I	Type IVb
CP628	A	M	N	A	D	A	D	F	P	A	I	Type IVb
CP281	A	M	N	A	D	A	D	F	P	A	I	Type IVb
CP458	A	M	N	A	D	A	D	F	P	A	I	Type IVb
CP457	A	M	N	A	D	A	D	F	P	A	I	Type IVb
CP428	A	M	S	A	D	A	D	F	P	A	I	Type IVc
CP438	A	M	S	A	D	A	D	F	P	A	I	Type IVc
CP450	A	M	S	A	D	A	D	F	P	A	I	Type IVc
CP157	A	M	N	D	D	A	D	F	P	A	I	Type IVd
CP714	A	L	N	A	A	T	D	F	P	A	I	Type V
CP885	A	L	N	A	A	T	D	F	P	A	I	Type V
CP616	A	L	N	A	A	T	D	F	P	A	V	Type Vd
CP672	A	L	N	A	A	T	D	F	P	A	V	Type Vd
CP792	A	L	N	A	A	T	D	F	P	A	V	Type Vd
CP667	A	L	N	A	A	T	D	F	P	A	V	Type Vd
CP489	A	L	N	A	A	T	D	F	P	V	V	Type Ve
CP88	A	L	N	A	A	T	D	F	P	V	V	Type Ve
CP412	A	L	N	A	A	T	D	F	P	V	V	Type Ve
CP757	A	L	N	A	A	T	D	F	P	V	V	Type Ve
CP734	A	L	N	A	A	T	D	F	P	V	V	Type Ve
CP666	A	L	N	A	A	T	D	F	P	V	V	Type Ve
CP833	A	L	N	A	A	T	D	F	P	V	V	Type Ve
CP832	A	L	N	A	A	T	D	F	P	V	V	Type Ve
CP782	A	L	N	A	A	T	D	F	P	V	V	Type Ve
CP706	A	L	N	A	A	T	D	F	P	V	V	Type Ve
CP91	A	L	N	A	A	T	D	F	P	V	V	Type Ve
CP396	A	L	N	A	A	T	D	F	P	V	V	Type Ve
CP859	A	L	N	A	A	T	D	F	P	V	V	Type Ve
CP301	A	L	N	A	A	T	D	F	P	V	V	Type Ve
CP232	A	L	N	A	A	T	D	F	P	V	V	Type Ve
CP94	A	L	N	A	A	T	D	F	P	V	V	Type Ve
CP570	A	L	N	A	A	T	D	F	P	V	V	Type Ve
CP502	A	L	N	A	A	T	D	F	P	V	V	Type Ve
CP228	A	L	N	A	A	T	D	F	P	V	V	Type Ve
CP327	A	L	N	A	A	T	D	F	P	V	V	Type Ve
CP422	A	L	N	A	A	T	D	F	P	V	V	Type Ve
CP400	A	L	N	A	A	T	D	F	S	A	I	Type Vf
CP653	A	L	N	A	A	T	Y	I	S	A	I	Type Vg
CP452	T	L	N	A	D	A	D	F	P	A	V	Type VIII

Alpha-toxin (Plc) of human *C. perfringens* strain 13 [16, 19] is shown as prototype to compare with Plc sequences of other strains, and described as "Type F" representing "first type". Amino acid substitutions compared with those of strain 13 are shown in yellow and blue, which denote those defining toxin type I, IV, V, and VIII reported previously [15], and those newly detected in the present study, respectively. 373I shown in gray indicates that this residue (isoleucine) is distinct from that in toxin type IV and IVa (valine) described by Abildgaard et al.[16] .

Table S2 *cpb2* nucleotide sequence identity (%) (upper right) and beta2 toxin amino acid sequence identity (lower left)

cluster	Strain	Sequence identity with strain								
		CP301	AN4361	AG07-9	CP412	CWC245	JGS1902	CP458	D13122	EGY2014
1	CP301		98.3	98.9	94.0	94.4	72.6	71.9	74.6	74.8
	AN4361	98.3		100.0	91.7	93.1	73.9	73.7	73.5	74.2
	AG07-9	98.7	100.0		94.1	94.9	73.8	74.7	73.8	74.1
prototype	CP412	90.2	87.2	92.1		97.7	71.3	71.3	73.7	74.8
	CWC245	91.7	88.4	92.7	96.6		71.7	71.0	73.6	74.8
2	JGS1902	63.6	69.2	67.5	61.0	62.5		97.4	97.2	95.9
	CP458	64.8	69.2	71.8	63.4	63.4	96.5		99.3	98.5
	D13122	68.1	68.6	67.5	66.4	66.4	95.6	98.9		99.0
	EGY2014	68.3	69.7	68.1	68.3	68.3	93.3	96.7	97.6	

Sequence identity within a same cluster is shown in yellow (nucleotide) or pink (amino acid).

Table S3 Primers used for PCR in the present study

Target gene	Primer	Nucleotide sequence (5'-3')	the other primer for PCR
cpe	cpe-5p1	GTCCATGTAGAAATATATTCAAG	+cpe-3p1, +cpe3p2
	cpe-3p1	GTAATATTAAATTATTACATAC	
	cpe-3p2	GAAAAATATTATCGTGATATATG	
bec (*cpile*)	becA-5p1	CATTTTTAAAAATGGAGGTAAAATATG	+bec-3p1, +becBR
	becB-3p1	GATATTTATATATTAAAATTAAAAAGGG	+bec-AF
	bec-BR	AGATTGGAGCAGAGCCAGAA	
	bec-AF	CAATGGGGCGAAGAAAATTA	+becB-1, +bec-3p1
	becB-1	CTTTTCCCCTTGATGGATCATC	+bec-BF
	bec-BF	TGCAAATGACCCTTACACTGA	
cpb2	cpb2-F2	ATGAAAAAAATTATTTCAAAG	+cpb2-R2
	cpb2-R2	CTTCATATTTTTTTCTATGCAC	
	cpb2-R	GCAGAATCAGGATTTTGACCA	+cpb2-L, +cpb2-L2
	cpb2-L	CAAGCAATTGGGGGAGTTTA	
	cpb2-L2	AGTCCAATGAAAGCAAGTGC	
	cpb2-F4	TTCAGGGGGGAATATAAATG	+cpb2-R3
	cpb2-R3	TTAGTTGTTGGTTAGGATCA	
plc	plc-F1	TTCAGTGCAAGTGTTAATCG	+plc-R1, +plc-R2, +plc-R4
	plc-R1	GCTTTTATTTTGTAAATACCACC	
	plc-R2	CTTTCCTCTGCAAAAGTCTCA	
	plc-R4	CTTTTATTATTTTATATTATAAGTTG	
	plc-F2	GGCTATGCACTATTTTGGAG	+plc-R1, +plc-R3, +plc-R4
	plc-R3	CCAAAACCAATAATTATTTTTTTAC	
netB	netB-5p1	GACCAGTTTTACCAAAGTTATTG	+netB-3pR1
	netB-3pR1	CTTTGATTAACTTGTAAGAAATC	

Table S4 GenBank accession numbers assigned for *C. perfringens* toxin genes analyzed in the present study

Strain ID	gene (s)				
	cpb2	*cpe*	*plc*	*bec* (*cpile*)	*netB*
CP157			MH900560		
CP228			MH900561		
CP301	MH900541				
CP322			MH900563		
CP327			MH900562		
CP396	MH900543	MH900564			
CP400			MH900556		
CP412	MH900542				
CP428	MH900548				
CP438	MH900544				
CP450	MH900545		MH900558		
CP452	MH900546		MH900551		
CP457	MH900547		MH900559		
CP458	MH900549				
CP606	MH920601		MH900552		
CP648	MH900550				
CP653			MH900557	MH900565	
CP672			MH900554		
CP832			MH900555		
CP885			MH900553		
CP859		MK118949			
CP201			MK902768		MK902770
CP238			MK902769		MK902771
